# Prolonged effect of antibiotic therapy on the gut microbiota composition, functionality, and antibiotic resistance genes’ profiles in healthy stool donors

**DOI:** 10.3389/fmicb.2025.1589704

**Published:** 2025-05-09

**Authors:** Ramin Karimianghadim, Reetta Satokari, Sam Yeo, Perttu Arkkila, Dina Kao, Sepideh Pakpour

**Affiliations:** ^1^School of Engineering, University of British Columbia, Kelowna, BC, Canada; ^2^Human Microbiome Research Program, Faculty of Medicine, University of Helsinki, Helsinki, Finland; ^3^Department of Gastroenterology, Helsinki University Hospital and Helsinki University, Helsinki, Finland; ^4^Division of Gastroenterology, Department of Medicine, Faculty of Medicine and Dentistry, University of Alberta, Edmonton, AB, Canada

**Keywords:** antibiotic treatment, fecal microbiota transplant, gut microbiome, antibiotic resistant genes, FMT donors

## Abstract

**Introduction:**

Fecal microbiota transplantation (FMT) is highly effective in preventing *Clostridioides difficile* recurrence by restoring gut microbiota composition and function. However, the impact of recent antibiotic use, a key exclusion criterion for stool donors, on gut microbiota recovery is poorly understood.

**Methods:**

We investigated microbial recovery dynamics following antibiotic use in three long-term stool donors from Canada and Finland. Using longitudinal stool sampling, metagenomic sequencing, and qPCR, we assessed changes in bacterial diversity, community composition, microbial functions, the gut phageome, and the risk of transmitting antibiotic-resistant genes (ARGs).

**Results:**

Antibiotics caused lasting disruption to bacterial communities, significantly reducing important taxa like *Bifidobacterium bifidum*, *Blautia wexlerae*, *Akkermansia muciniphila*, *Eubacterium* sp. *CAG 180*, and *Eubacterium hallii*, with effects persisting for months. Functional analyses revealed alterations in housekeeping genes critical for energy production and biosynthesis, with no direct links to key health-related pathways. Antibiotics also disrupted viral populations, decreasing diversity and increasing *crAssphage* abundance, reflecting disrupted host-bacteriophage dynamics. No significant increase in clinically important ARGs was detected.

**Discussion:**

These findings highlight the unpredictable and complex recovery of gut microbiota post-antibiotics. Individualized suspension periods in donor programs, guided by metagenomic analyses, are recommended to optimize FMT outcomes in various indications by considering antibiotic spectrum, duration, and host-specific factors.

## 1 Introduction

Fecal Microbiota Transplantation (FMT) is highly effective in managing recurrent *Clostridioides difficile* infection (rCDI), achieving success rates of 80 to 90%, and is recommended by multiple practice guidelines (Van Nood, 2013; McDonald et al., 2018; Ng et al., 2020; Cammarota et al., 2019; Haifer et al., 2020). FMT has also shown promise in several other dysbiosis-associated conditions, including inflammatory bowel disease (IBD) (Paramsothy et al., 2017; Fischer et al., 2016; Moayyedi et al., 2015), metabolic syndrome (Mocanu et al., 2021), irritable bowel syndrome (Holvoet et al., 2021), multidrug-resistant bacteria de-colonization (Battipaglia et al., 2019; Bilinski et al., 2017), and immune checkpoint inhibitor-associated colitis (Wang et al., 2018). Presently, there are approximately 572 registered clinical trials worldwide (WHO, 2023), exploring FMT’s therapeutic potential across these diverse conditions (ClinicalTrials.gov, 2023).

Although the mechanisms of action underpinning the therapeutic efficacy of FMT remains incompletely understood, bacterial engraftment is thought to be crucial (Podlesny et al., 2022). While pathogen-free donor stool is essential, specific microbiota composition may be less critical given high success rates (Van Nood, 2013; Cammarota et al., 2015; Kassam et al., 2013). However, for conditions with complex pathophysiology, the therapeutic efficacy of FMT may be closely tied to the diversity, specific microbial taxa, and functionalities provided by the donor microbiota (Paramsothy et al., 2017).

Antibiotics disrupt the gut microbiota by reducing microbial diversity, eliminating individual taxa, with changes that can persist for months or even years. Moreover, recovery is not solely about microbial diversity; functional stability is critical, given that specific microbial activities are governed at the strain level. While diversity may rebound relatively quickly, certain beneficial strains and their associated functions may take much longer to fully recover (Dethlefsen and Relman, 2011; Lozupone et al., 2012; Palleja et al., 2018). Another significant concern with antibiotic exposure in FMT donors is the enrichment of antibiotic-resistant microbes and increased risk for transmission of antibiotic-resistant genes (ARGs) to the recipient’s microbiota (Strati et al., 2021), posing long-term health risks if resistance genes transfer to pathogenic strains. The persistence of ARGs in the donor microbiota can occur even after apparent recovery of microbial diversity.

Balancing safety and efficacy remains particularly challenging in non-rCDI cases, where donor selection can substantially influence therapeutic success (Liptak et al., 2021). While the efficacy of FMT for rCDI is well-established, rare but severe complications, such as transmission of multi-drug resistant organism resulting in death has been reported, emphasizing the importance of meticulous donor screening to avoid transmitting pathogens or ARGs, particularly in immunocompromised individuals (Food and Drug Administration, 2020, 2022, 2023; van Lingen, 2023).

Current guidelines recommend a three-month suspension period post-antibiotic use (Kelly et al., 2016; Keller et al., 2021; Mullish et al., 2018). However, this three-month threshold is not strongly evidence-based and stems from limited studies. This is concerning, as key bacterial taxa essential for various physiological functions may take significantly longer to return to baseline levels (Dethlefsen and Relman, 2011; Palleja et al., 2018; Raymond et al., 2016), highlighting a gap in donor requalification practices (Dethlefsen and Relman, 2011; Jernberg et al., 2007; Korpela et al., 2016; Ng et al., 2019). Notably, a recent study demonstrated that antibiotic use by donors within 3–12 months prior to donation significantly decreased FMT effectiveness (Grosen et al., 2025). Therefore, determining an optimal antibiotic-free interval is essential to ensure that the donor microbiota has adequately recovered in both diversity and function to support FMT efficacy across a range of clinical applications. This study aims to investigate the effects of antibiotic exposure on donor microbiota composition, functional stability, and the persistence of ARGs using advanced high-throughput metagenomic sequencing. By clarifying microbiome recovery dynamics in real-life examples of fecal donors who received antibiotic therapy and were subsequently quarantined from donating, this research seeks to improve donor screening practices, and inform evidence-based guidelines that enhance both the safety and therapeutic efficacy of FMT.

## 2 Materials and methods

### 2.1 Sample collection and DNA extraction

**Figure 1:**
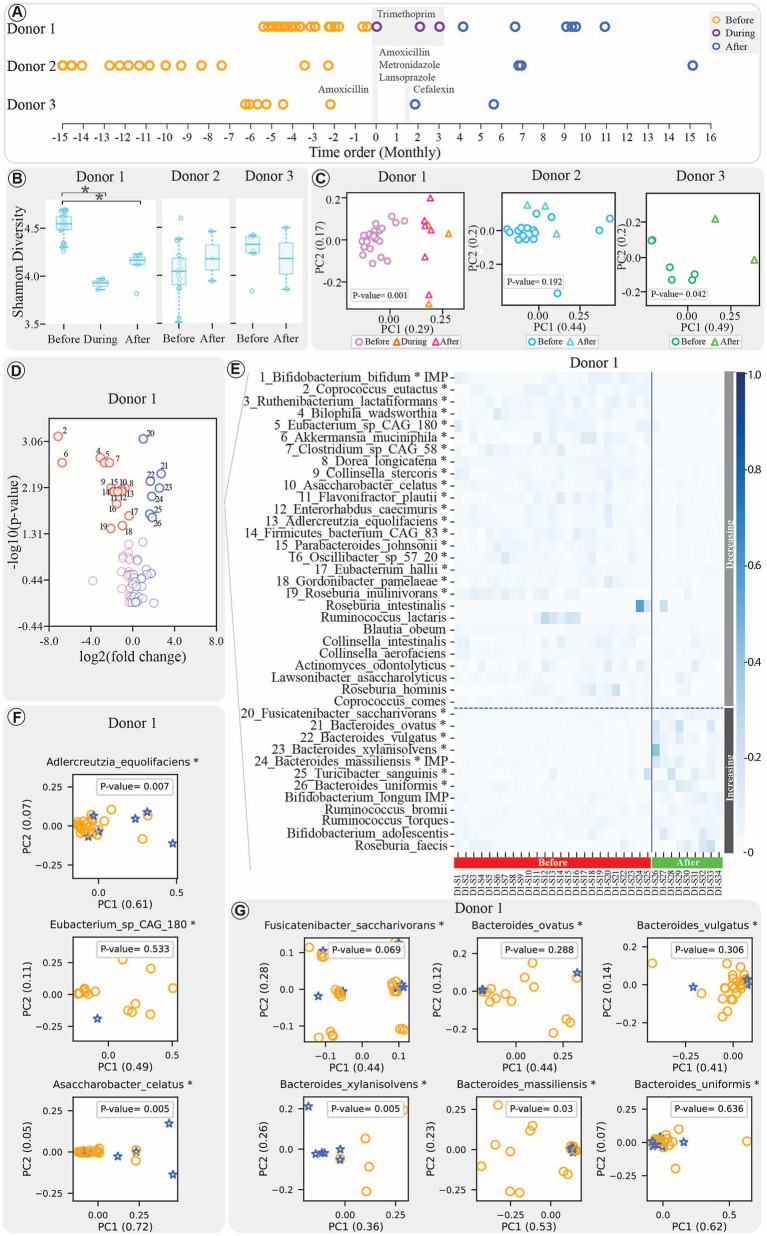
**(A)** Donor samples before, during (for Donor 1), and after treatment are represented by the orange, purple, and blue colors, respectively. The x-axis reflects a one-month time interval, with donor sampling dates rescaled to facilitate direct comparisons. All timelines are standardised so that antibiotic therapy begins at month 0 for each donor, despite their collection at various time periods. The shaded gray regions represent the duration of the specific treatment applied to each donor, with the type of antibiotics indicated in the figure for each donor. **(B)** Boxplots comparing the Shannon diversity index of samples’ bacteriome profile before, during (for Donor 1), and after treatment. (*indicates significant change with FDR-adjusted *p*-value < 0.05). **(C)** PCoA plots comparing beta diversity of samples before, during (for Donor 1), and after treatment for each donor, with *p*-values displayed within the figure. **(D)** Volcano plot displaying bacterial species’ response to treatment. The x-axis shows log₂ change in feature abundances before vs. after treatment; the y-axis shows -log₁₀ of the adjusted *p*-value. Significant changes (FDR-adjusted *p*-value < 0.05, Mann–Whitney) are indicated in the figure. **(E)** Heatmap illustrating dynamics of the top 40 bacteria (sorted by raw *p*-values), with horizontal dashed line separating decreasing and increasing bacteria, and solid vertical line distinguishing before and after treatment samples (*indicates significant change with FDR-adjusted *p*-value < 0.05, Mann–Whitney test). The samples are presented in chronological order in this figure. **(F,G)** PCA plots of strain communities with significant decrease **(F)** or increase **(G)** post-treatment, using samples meeting the StrainPhlan pipeline’s minimum thresholds (details in Methods). Circles and stars denote pre- and post-treatment samples, respectively.

Stool samples were collected longitudinally from three individuals before and after antibiotic treatments. Ethics approval for the use of donor samples in this study was obtained (refer to the Ethics Approval section for details). Donor 1 (Alberta, Canada) provided 34 samples over a 31-month period between June 25, 2019, and January 15, 2022, during which he underwent a 3-month Trimethoprim treatment. Donor 2 (Helsinki, Finland) provided 19 samples over a 19-month period between May 29, 2012, and December 19, 2014, and was exposed to a one-week *Helicobacter pylori* (*H. pylori*) eradication therapy (amoxicillin 500 mg four times a day, metronidazole 400 mg three times a day, and lansoprazole 30 mg twice a day). Lastly, Donor 3 (Helsinki, Finland) provided 8 samples over a 13-month period between May 29, 2012, and June 1, 2013, and underwent two antibiotic treatments: a one-week amoxicillin regimen (500 mg three times per day) followed by a one-week cefalexin regimen (500 mg twice per day). The timeline of all three donors’ samples is depicted in Figure 1A. All stool samples were aliquoted and stored at −80° C within 4 h of stool collection. The donors were quarantined from acting as FMT donors for at least 3 months after the antibiotic exposure.

Microbial DNA from stool samples was extracted using the FastDNA Spin Kit for Feces (MP Biomedicals, Solon, Ohio) for whole genome shotgun sequencing. Metagenome libraries were constructed with the Nextera XT protocol (developed by Illumina) and sequenced on an Illumina MiSeq platform using a paired-end 300 cycle protocol.

### 2.2 Sequencing and bioinformatics

The preprocessing of metagenomic sequencing data aimed to ensure high-quality data for downstream analysis. The KneadData^1^ pipeline (v0.10) was used to preprocess raw reads, employing Trimmomatic (v0.39), Bowtie2 (v2.3.5.1), and custom scripts to remove low-quality reads, primers, and sequencing adapters, and host contamination from the input sequencing data. Adjustments to default settings included SLIDINGWINDOW:4:25, HEADCROP:10, and MINLEN:77. Post-processing, an average of 25 million reads per sample was obtained, with a read length of 131 base pairs and a quality score above 35, providing sufficient sequencing depth.

The bacterial community composition was analyzed using MetaPhlan3 (v3.0.14) and Kraken2 (v2.1.2), and their performances were compared (Beghini et al., 2021; Wood et al., 2019). MetaPhlan3 was run with the “-t rel_ab_w_read_stats” parameter, mapping quality-controlled reads to a marker gene database (v30^2^) with Bowtie2. Post-processing involved removing low-abundance taxa (<0.01%), singletons, doubletons, and species found in fewer than half of the samples, resulting in 72 distinct bacterial species. Kraken2 was run with a standard database (March 13, 2022) and specific parameters (-kmer-len: 35, -minimizer-len: 31, and -minimizer-spaces: 7). The read count profile for each sample was normalized by dividing each value by the total sequence count in that sample. Post-processing involved filtering singletons, doubletons, and bacteria below 1, 0.1, or 0.01% abundance, followed by the exclusion of species present in fewer than half of the samples.

Microbial strain analysis focused on characterizing strain-level variations in bacterial species. The StrainPhlAn pipeline was used in this regard (v4.0.3; Truong et al., 2017). Samples with at least 20 markers were retained (−sample_with_n_markers), and markers present in at least 50% of the samples were kept (−marker_in_n_samples). StrainPhlAn then categorized these samples as distinct strains based on the distance between strain sequences. This distance was used to create a distance matrix for PCoA plots, implemented with the Scikit-bio^3^ Python library (v0.5.6). Strain analysis was conducted only on species that showed significant changes in the study.

For virome analysis, the same Kraken2 pipeline and database settings as used for bacteriome analysis were applied. Kraken2 output was filtered to include only viruses, and post-processing normalized read counts per sample by dividing by the total number of sequences. Singletons, doubletons, viruses with abundances below 0.01%, and viruses found in fewer than half of the samples were removed. After processing, 14 distinct viral species remained, accounting for nearly 96% of the viral reads. It is important to note that our virome analysis exclusively detects DNA viruses, as RNA viruses would require complementary RNA-specific extraction and sequencing methods.

*CrAssphage* abundance was assessed using two approaches: relative abundance from metagenomics and absolute abundance via qPCR. For relative abundance, *crAssphage* levels were extracted from Kraken2 virome analysis outputs. For absolute abundance of c*rAssphage*, qPCR assays were conducted using a 126 bp gBlock gene fragment (crAssphage CPQ_056 amplicon) as a standard (Stachler et al., 2017). Calibration curves were created with 10-fold dilutions from 10 ng/μL to 10^−7^ ng/μL of the gBlock. qPCR was performed in triplicate with Itaq™ Universal Probes Supermix, primers, and probe, using the CFX Opus 96 instrument. DNA concentrations were converted to genomic copies/μL, as described in (Stachler et al., 2017), which provides additional details of the qPCR assays. Also, detailed information regarding primer sequences and qPCR cycling conditions is provided in Supplementary Table S1.

The functional potential of bacterial communities was assessed using the Humann3 pipeline (v3.6) by profiling the pathway abundances in samples (Beghini et al., 2021). Default parameters were applied, and pathway abundances were normalized using the TSS approach with the “--units relab” option for relative abundances. The post-processing steps were applied by removing singletons, doubletons, pathways below 0.01% abundance, and those present in fewer than half of the samples. This resulted in a final set of 286 unique pathways.

To investigate the ARGs profiles of samples, the Comprehensive Antibiotic Resistance Database (CARD, v3.1.4) and its Resistance Gene Identifier (RGI, v5.2.1) program were used (Alcock et al., 2023). The “bwt” option for metagenomic short reads and Bowtie2 for alignment were employed. Read counts for mapped reads were analyzed at the gene level, and sample tables were merged using custom Python scripts. The obtained abundance table was subsequently subjected to a post-processing step to remove singletons, doubletons, and ARGs with abundances below 0.1%. The final table contained 121 unique ARGs.

### 2.3 Statistical and data analysis

For majority of the data analysis, Python (v3.9) packages and scripts were used, with visualization done by the Matplotlib package (v3.7.1; Hunter, 2007).

Stacked bar plots were created with Matplotlib (v3.7.1). Samples were rescaled by dividing abundances by the total sum per sample, and features (bacteria/ARGs) were sorted by their average values. Only a subset of highly abundant features was colored and shown in the legend.

Alpha diversity was assessed using the Shannon metric from Scikit-bio library (v0.5.6) and visualized with boxplots from Matplotlib library (v3.7.1). Statistical significance was determined using the Mann–Whitney U test from SciPy library (v1.7.3) with a significance threshold set at a *p*-value < 0.05 (Virtanen et al., 2020). For Donor 1, which includes three groups (before, during, and after antibiotic exposure), the Kruskal–Wallis test from the SciPy library was first applied to assess overall differences. This was followed by pairwise Mann–Whitney U tests for *post-hoc* analysis, with FDR correction applied to account for multiple comparisons. Beta diversity was analyzed with PCoA plots based on Bray-Curtis distances using the Scikit-learn library (Pedregosa et al., 2011). The comparison between the groups is performed using the PERMANOVA test from the Scikit-bio library, with a significance threshold set at a *p*-value < 0.05. For Donor 1, if the PERMANOVA test yielded a significant *p*-value, a *post-hoc* analysis (pairwise PERMANOVA with FDR correction) was conducted.

If no significant differences were detected between the during and after antibiotic exposure samples in Donor 1, these samples were combined and considered as after antibiotic exposure samples to increase the sample size for subsequent analyses.

For correlation network analysis, Spearman correlations between bacterial species and ARGs were calculated using SciPy (version 1.7.3). The correlation analysis included ARGs and only the significantly changed bacteria as well as bacteria important for FMT engraftment (Supplementary Table S2). Only positive correlations with FDR-adjusted *p*-values < 0.05 and R-values > 0.6 were retained. Networks were created with NetworkX (version 2.6.3) and visualized with Gephi (version 0.9.6), where node size reflects the significance of the change after treatment (Mann–Whitney U test, *p* < 0.05) and edge thickness indicates correlation strength. Green links represent correlations unique to post-treatment samples, which did not exist in before treatment samples.

For constructing the phylogenetic tree via the ETE Python library (v3.1.3; Huerta-Cepas et al., 2010), the union of all bacterial species (from all the samples) identified by either Kraken2 or MetaPhlan3, utilizing various filtering thresholds, is employed. The tree, annotated with metadata on species identification methods, was visualized using the Interactive Tree of Life (iTOL; v6.8; Letunic and Bork, 2019).

## 3 Results

### 3.1 Assessing gut microbial diversity and composition in donors before and after antibiotic treatment

In our investigation of gut microbiota, we aimed to understand how antibiotic exposure influences the composition and diversity of the gut bacteriome in donor samples. To ensure accurate bacterial species profiling, we chose the MetaPhlan3 pipeline over Kraken2 for its conservative approach to bacterial species identification. Supplementary Table S3 shows the number of unique species identified at various thresholds, with species coverage illustrated in Supplementary Figure S1.

Since the baseline gut microbiota may determine its ability to recover following perturbation, we compared the bacteriomes of donors before antibiotic treatment. Alpha diversity analysis revealed significant differences between Donor 1 and Donors 2 and 3 (Mann–Whitney U test with FDR correction, adjusted *p*-values < 0.0001 and < 0.01, respectively), while no significant difference was observed between Donors 2 and 3 (Supplementary Figure S2A). Beta-diversity analysis confirmed that each donor had a distinct microbial community, with significant differences across all donors (Pairwise PERMANOVA test with FDR correction, adjusted *p*-value < 0.01; Supplementary Figure S2B). Furthermore, bacterial composition at the phylum level (Supplementary Figure S2C) showed Firmicutes as the predominant phylum in all donors. Actinobacteria was the second most abundant in Donor 2, while Bacteroidetes was the second most abundant phylum in Donors 1 and 3. At the species level (Supplementary Figure S2D), *Faecalibacterium prausnitzii* dominated in Donors 1 and 3, while *Bifidobacterium adolescentis* and *Bifidobacterium longum* were prevalent in Donor 2.

Following antibiotic treatment, we observed significant changes in the microbial communities. Donor 1 showed a significant difference in alpha diversity across the before, during, and after antibiotic exposure groups (Kruskal–Wallis test, *p* < 0.0001). *Post-hoc* analysis in this donor revealed a significant decrease in alpha diversity in both the during and after treatment samples compared to before treatment group (Mann–Whitney U test with FDR correction, adjusted *p*-value < 0.001). Also, Donor 3 showed a non-significant decrease (Mann–Whitney U test, *p*-value = 0.42). In contrast, Donor 2, sampled 7–15 months post-treatment, exhibited a slight, non-significant increase in diversity (Mann–Whitney U test, *p*-value = 0.86; Figure 1B). Beta diversity analysis revealed significant compositional changes in Donors 1 and 3 (PERMANOVA test, *p*-value < 0.05), but not in Donor 2, whose microbial composition remained stable (PERMANOVA test, *p*-value = 0.208; Figure 1C). Furthermore, *post-hoc* analysis of Donor 1 samples revealed that the bacteriome composition of the during and after treatment samples was significantly different from the before treatment group (PERMANOVA test with FDR correction, adjusted *p*-value < 0.005), while no significant difference was observed between the during and after treatment communities (PERMANOVA test with FDR correction, adjusted *p*-value = 0.86).

A closer look at specific bacterial species showed that Donor 1 experienced significant shifts in 26 taxa, with 19 decreasing and 7 increasing (Mann–Whitney U test with FDR correction, adjusted *p*-value < 0.05; Figures 1D,E). Donor 2 exhibited fewer changes: *Blautia wexlerae* showing a significant decrease, and six taxa—including *Bacteroides fragilis* and *Bacteroides vulgatus*— showing significant increases (Mann–Whitney U test with FDR correction, adjusted *p*-value < 0.05; Supplementary Figures S3A,B). Donor 3’s bacterial landscape remained stable, with no significant changes post-treatment (Supplementary Figures S3D,E).

To gain a more detailed understanding, strain-level analysis was conducted using the StrainPhlAn pipeline. In Donor 1, although some species showed significant changes, the strain populations within these species remained relatively stable. For instance, among the bacteria with decreasing abundance, eg. *Eubacterium* sp. *CAG 180*, and among those with increasing abundance, eg. *Fusicatenibacter saccharivorans*, *Bacteroides ovatus*, *Bacteroides vulgatus*, and *Bacteroides uniformis,* have not shown significant strain population changes (Figures 1F,G, respectively). In Donor 2, strains of *Eubacterium sp_CAG 180* and *Bacteroides vulgatus* remained stable, while *Blautia obeum* exhibited significant strain changes post-treatment (PERMANOVA test, *p*-value < 0.05; Supplementary Figure S3C). Strain analysis was not performed for Donor 3 due to the lack of significant findings at the species level.

### 3.2 Phageome composition and *crAssphage* abundance in donor samples before and after antibiotic exposure

**Figure 2:**
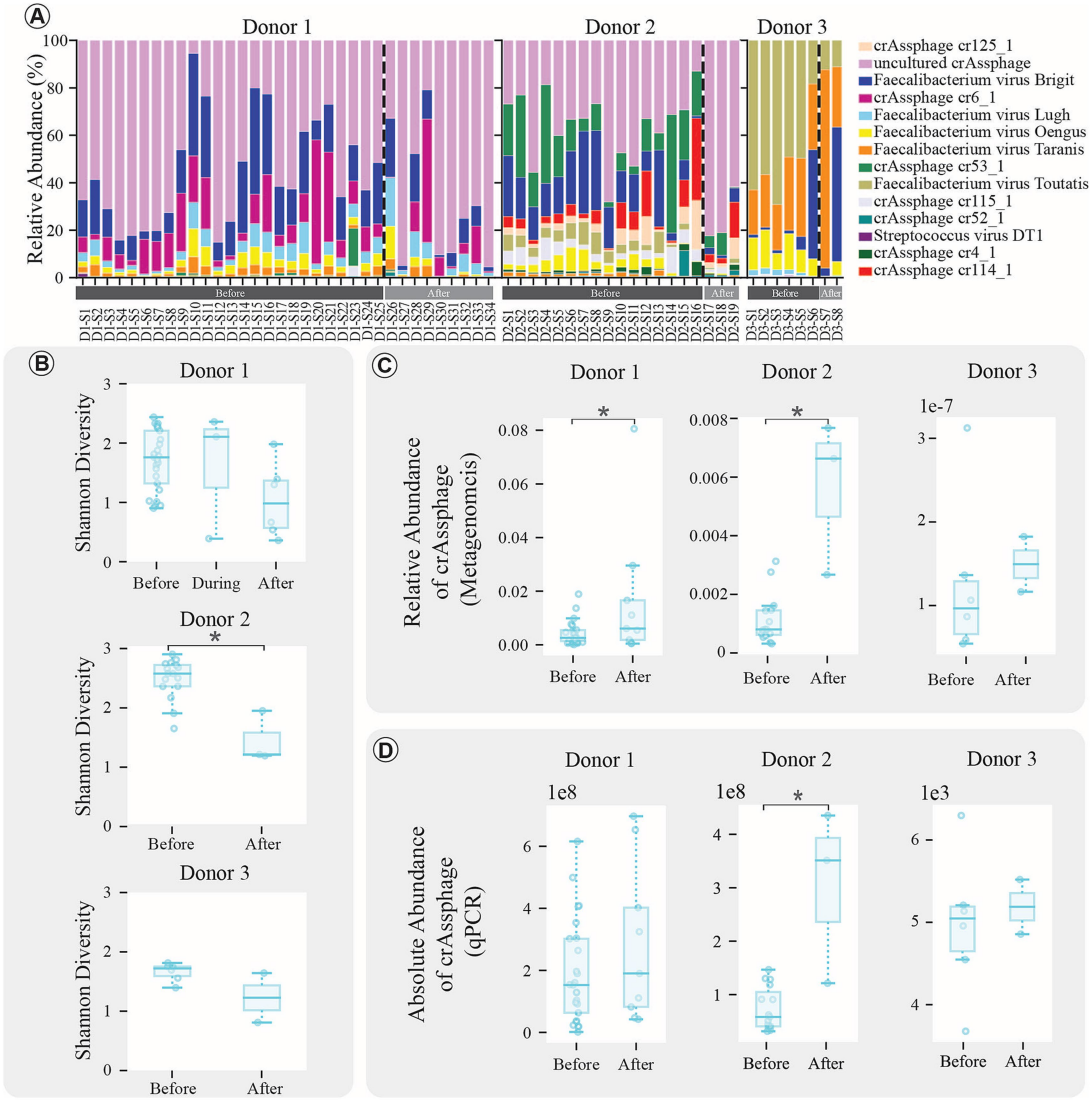
Virome composition analysis results. **(A)** Stacked bar plots depicting virome compositions at the species level. Before, during (for Donor 1), and after treatment samples are differentiated by a dashed line and the samples are presented in chronological order. **(B)** Boxplots comparing the Shannon diversity index of samples before, during (for Donor 1), and after treatment. (*indicates significant change with *p*-value < 0.05). **(C)** Boxplots comparing the relative abundance of *crAssphage* using the metagenomics data from Kraken2 pipeline (*indicates significant change with *p*-value < 0.05). **(D)** Boxplots comparing the absolute abundance of *crAssphage* using the qPCR approach data (*indicates significant change with *p*-value < 0.05).

To comprehend how antibiotic exposure influences the composition and diversity of the gut virome in donor samples, we examined the viral species composition of the donor samples. Overall, 14 viral species were identified belonging to the phylum Uroviricota. The viral species composition of Donors samples is illustrated using the stacked bar graphs in Figure 2A. Notably, the samples from Donor 3 exhibited a different virome composition compared to the other donors. In Donor 1 and Donor 2, the most abundant virus is “*uncultured_crassphage.”* However, it is intriguing that this virus was hardly detectable in Donor 3. Additionally, when examining the impact of antibiotic treatment on post-treatment sample diversity, a decrease in the alpha diversity of viruses was observed in all donors, as shown in Figure 2B. This decrease reached statistical significance only in Donor 2 (Mann–Whitney U test, *p*-value < 0.01), while no significant differences were observed between groups in Donor 1 (Kruskal–Wallis test, *p* = 0.085) or Donor 3 (Mann–Whitney U test, *p* = 0.285).

Since *CrAssphage* is known as a dominant phage in the human gut microbiota, it was taken into further investigation. The results demonstrated that total *crAssphage* abundance increased following antibiotic treatment, with statistical significance observed in Donor 1 and Donor 2 (Mann–Whitney U test, *p*-value < 0.05; Figure 2C). The findings were validated by qPCR to obtain absolute abundance measurements for *crAssphage*. The qPCR results supported the increase in *crAssphage* abundance after antibiotic therapy, reaching statistical significance in Donor 2 (Mann–Whitney U test, *p*-value < 0.05; Figure 2D). Standard curves for each donor showed high reliability with R-squared values near 1, indicating robust qPCR assays across all donors, further supporting the validity of these findings (Supplementary Figure S4).

Beyond the antibiotic impact on the gut virome, we explored potential correlations between *crAssphage* abundance, bacterial species, and ARGs. We hypothesized that *crAssphage* abundance might be linked to alterations in the bacterial community and ARGs. The analysis, conducted using qPCR results to avoid autocorrelation issues from metagenomics data, did not reveal any significant correlations between *crAssphage* abundance and specific bacterial species or ARGs (Spearman correlation, *p*-value < 0.05, R-value < 0.6).

### 3.3 Functional changes in the gut microbiome post-antibiotic treatment

**Figure 3:**
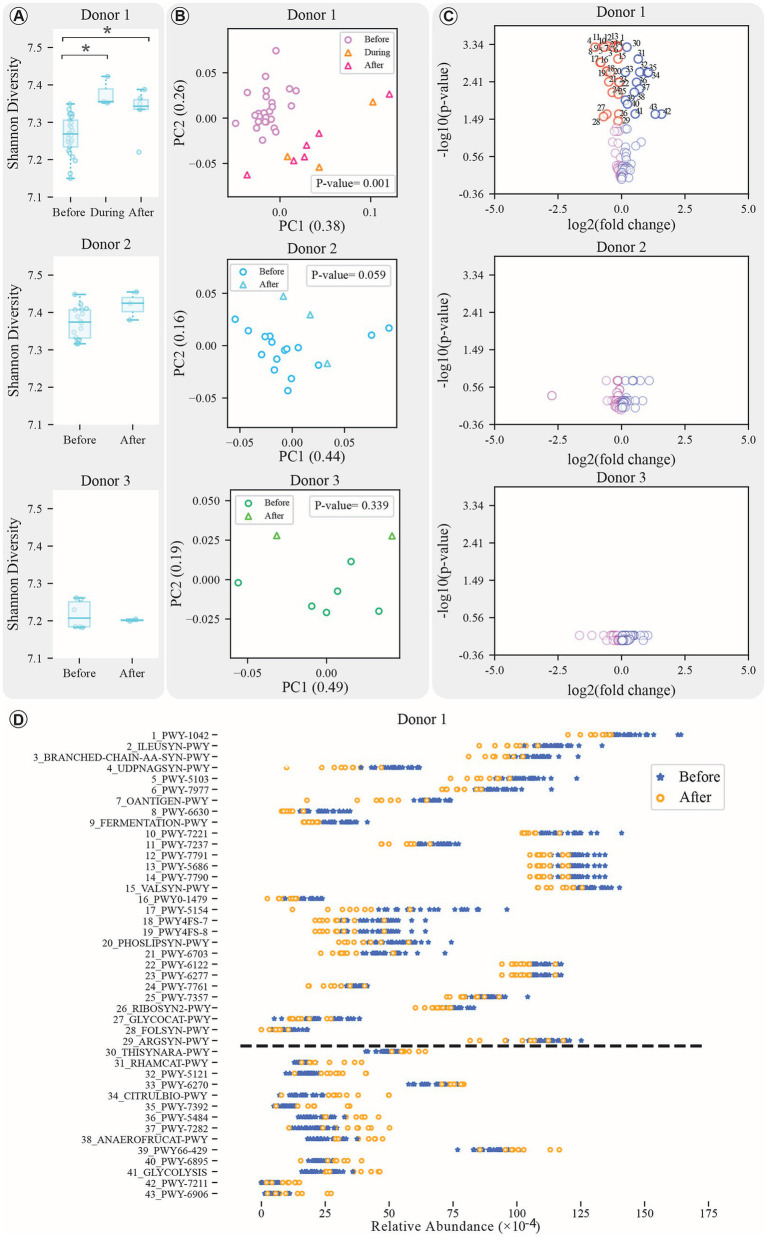
Pathway analysis results. **(A)** Boxplots comparing the Shannon diversity index of samples before, during (for Donor 1), and after treatment for all donors (*indicates significant change with *p*-value < 0.05). **(B)** PCoA plots comparing beta diversity of samples before, during (for Donor 1), and after treatment for each donor, with *p*-values displayed within the figures. **(C)** Volcano plot of pathways’ response to treatment. The x-axis shows log₂ change in feature abundances before vs. after treatment; the y-axis shows -log₁₀ of the adjusted *p*-value. Only the top 100 pathways contributing to PCA components are shown. Significant changes (FDR-adjusted *p*-value < 0.05, Mann–Whitney) are indicated in the figure. **(D)** Dynamics of the significantly changed pathways for Donor 1 (increased and decreasing pathways are separated using a dashed line).

To explore how antibiotic exposure affects the functional genes within the donor microbiota, we analyzed changes in pathway abundance profiles. Our analysis revealed that only Donor 1 experienced significant changes in both alpha and beta diversity during and after treatment compared to pre-treatment phase (Figures 3A,B; Kruskal–Wallis test, *p*-value < 0.05; Mann–Whitney U test and PERMANOVA test, FDR-adjusted *p*-value < 0.05). In contrast, Donors 2 and 3 showed no substantial alterations (Figure 3C). Moreover, in Donor 1, no significant difference was observed between during and after treatment groups (Mann–Whitney U test, FDR-adjusted *p*-value = 0.262).

In Donor 1, 43 pathways were significantly altered after antibiotic exposure, 29 decreased and 14 increased (Supplementary Table S4). These pathways span 13 unique super classes, with the most frequently decreased ones including “Amino Acid Biosynthesis” (8 times), “Nucleoside and Nucleotide Biosynthesis” (6 times), and “Cofactor, Carrier, and Vitamin Biosynthesis” (4 times). Among the increasing groups, “Secondary Metabolite Biosynthesis” appeared most frequently (3 times). Within these categories, certain pathways exhibited significant decreases and increases following treatment, encompassing a variety of metabolic processes, biosynthesis pathways, and degradation processes (Mann–Whitney U test with FDR correction method, adjusted *p*-values < 0.05; Figure 3D; Supplementary Table S4).

The pathway analysis revealed that bacterial contributions to these pathways come not only from significantly altered species but also from those without significant changes, contributing at least 5% to certain pathways (Supplementary Figures S5, S6). For example, the “O-antigen building blocks biosynthesis” pathway (OANTIGEN-PWY) involves *Bifidobacterium adolescentis* and *Collinsella aerofaciens*, while the “thiamine diphosphate biosynthesis III” pathway (THISYNARA-PWY) is influenced by both significantly altered and stable bacteria, such as *Bacteroides uniformis*, *Ruminococcus torques*, *Faecalibacterium prausnitzii*, and *Roseburia faecis.*

A significantly increased bacterium, *Bacteroides vulgatus*, has contributed to both the reduction and increase of several key pathways: PWY-7977, PWY-1042, PWY-6703, and RIBOSYN-PWY among the decreased pathways, and PWY66-429, RHAMCAT-PWY, and PWY-5121 among the increased pathways (Supplementary Figures S5, S6). *Bifidobacterium bifidum*, significantly decreased post-treatment, contributed to the reduced “glycogen degradation I” pathway (GLYCOCAT-PWY; Supplementary Figure S5). These findings highlight the complex interplay between bacterial species—both significantly altered and stable—and their collective influence on pathway dynamics.

### 3.4 Diversity and dynamics of ARGs and their species associations post-antibiotic treatment

**Figure 4:**
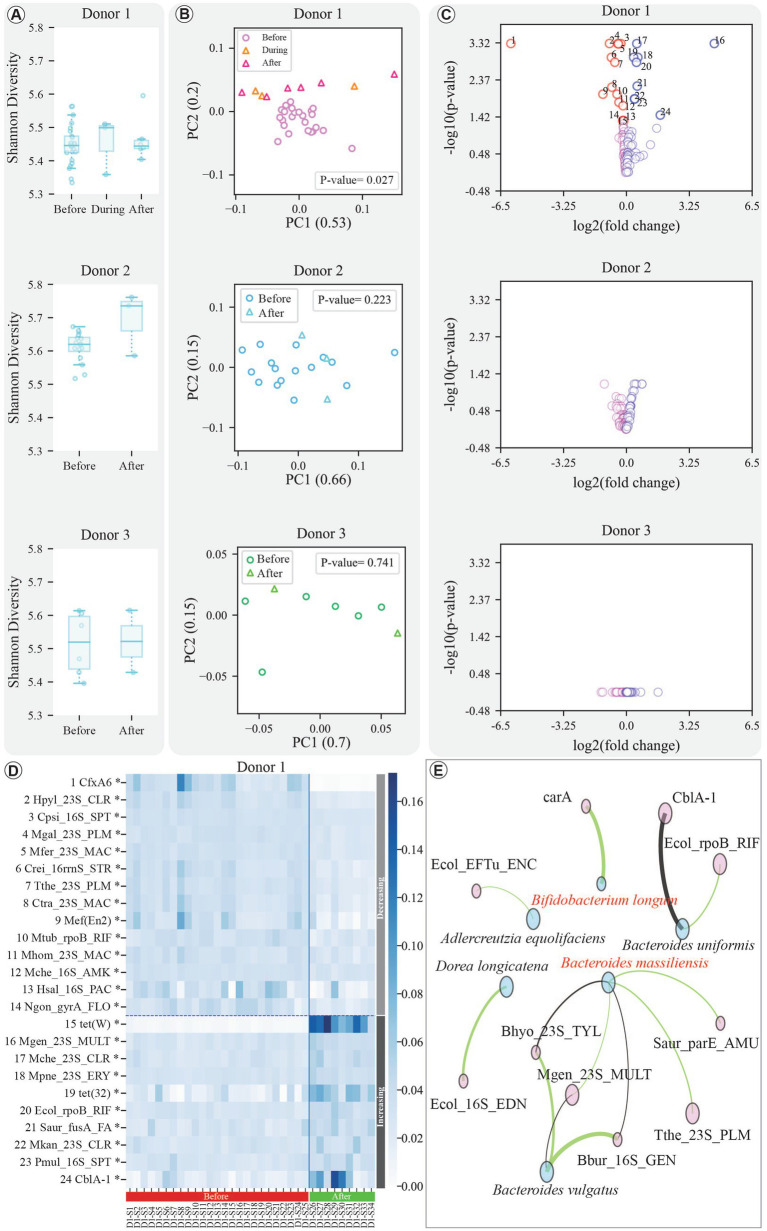
Results of ARG analysis. **(A)** Comparative Boxplots illustrating the Shannon diversity index of samples before, during (for Donor 1), and after treatment across all donors. **(B)** PCoA (Principal Coordinates Analysis) plots depicting the beta diversity of samples before, during (for Donor 1), and after treatment for each, with *p*-values displayed within the figures. **(C)** Volcano plot of ARGs’ response to treatment. The x-axis shows log₂ change in feature abundances before vs. after treatment; the y-axis shows -log₁₀ of the adjusted *p*-value. Significant changes (FDR-adjusted *p*-value < 0.05, Mann–Whitney) are indicated in the figure. **(D)** Heatmap displaying the dynamics of significantly altered ARGs for Donor 1. The heatmap differentiates between increased and decreased pathways using a horizontal dashed line, and before-and-after treatment samples are separated by a vertical solid line. The samples are presented in chronological order in this figure. **(E)** Correlation network illustrating positive and significant links among significantly changed bacterial species (as well as the crucial bacteria for FMT engraftment, highlighted in red text) and ARGs. The network considers post-treatment samples and employs correlation *p*-value and R-value thresholds of 0.05 and 0.6, respectively, using the Spearman correlation method (details in Methods). Node size corresponds to the magnitude of change after treatment, with larger nodes indicating significantly changed bacteria or ARGs after treatment.

The profiles of ARGs were analyzed to determine whether antibiotic exposure leads to changes in the abundance and prevalence of ARGs within the donor microbiota, assessing potential increased risks of transfer in FMT. The results revealed that while overall alpha diversity of ARGs showed no significant changes across donors (Kruskal–Wallis test and Mann–Whitney U test, *p*-value > 0.05; Figure 4A), beta diversity revealed distinct patterns (Figure 4B). Donor 1, in particular, exhibited a significant shift in ARG community composition post-treatment (PERMANOVA test, FDR-adjusted *p*-value < 0.01), while no significant difference was observed between the during and after treatment groups (Mann–Whitney U test, FDR-adjusted *p*-value = 0.090). The full names of the significantly changed ARGs for Donor 1 are listed in Supplementary Table S5.

As the composition of post-treatment samples in Donor 1 significantly differed from the pre-treatment samples, the dynamics of ARG abundance in this donor were further investigated. The post-treatment ARG abundance for this donor revealed significant increases and decreases in several ARGs, visualized in a volcano plot and heatmap (Figures 4C,D; Mann–Whitney U test with FDR correction, adjusted *p*-value < 0.05).

Association mapping identified several noteworthy correlations between ARGs and specific microbial taxa (Figure 4E). For example, the Mgen_23S_MULT gene showed a significant positive association with *Bacteroides massiliensis* (Spearman correlation with FDR correction, adjusted *p*-value < 0.05, R-value > 0.6). Similarly, *Bifidobacterium longum*, was positively correlated with carA (adjusted *p*-value < 0.01, R-value > 0.6). Also, *Bacteroides vulgatus* (currently *Phocaeicola vulgatus*), exhibited new associations with ARGs such as Bbur_16S_GEN and Bhyo_23S_TYL (adjusted *p*-values < 0.01 and < 0.05, respectively). Other positive correlations are shown in Figure 4E.

## 4 Discussion

While the efficacy of FMT at preventing CDI recurrence is well established, the duration of donor suspension following a course of antibiotic is under-investigated. Our analysis highlights differences in recovery in three long term stool donors in microbial diversity, functionality, phage abundance and diversity, as well as ARG profiles. The most notable disruptions were seen in Donor 1, who underwent a longer course of antibiotics and showed significant changes in microbial diversity, composition, functionality, and ARG profiles that persisted even 8 months post-treatment. In contrast, Donor 2 exhibited relative compositional stability between 7 and 15 months after treatment. These findings suggest that in cases of prolonged antibiotic exposure, microbiota recovery may take up to 8 months or longer. Conversely, for shorter antibiotic courses, an 8-month window may represent a reasonable timeframe for microbiota recovery and donor requalification.

Studies have shown that several engrafted bacteria during FMT treatment, especially from the Bacteroidetes and Actinobacteria phyla, are closely linked to beneficial functions like SCFA and bile acid metabolism (listed in Supplementary Table S2) Ojima et al., 2020; Morrison and Preston, 2016; Harris et al., 2018; Smith et al., 2022; Smillie et al., 2018; Kootte et al., 2017; Ianiro et al., 2022). Our study revealed that antibiotic exposure leads to reduction or near complete disappearance of specific SCFA producers like *Bifidobacterium bifidum*, *Roseburia inulinivorans*, *Eubacterium hallii*, and *Akkermansia muciniphila*. In one donor, this disruption extended beyond microbiota composition, affecting metabolic pathways, including the biosynthesis of essential amino acids like L-arginine and L-methionine, which are particularly underrepresented in IBD patients (Hellmann et al., 2020; Li et al., 2023). We also observed alterations in the mixed acid fermentation pathway, which is associated with acetate production (Förster and Gescher, 2014). Even when the abundance of some bacteria remains relatively unchanged, shifts in their strain communities, as seen with *Bacteroides massiliensis* and *Bacteroides xylanisolvens*, may still impact their functionality. These strain-level shifts underscore the complexity of microbial recovery following antibiotic exposure, as even subtle variations in strain composition can lead to significant changes in functional capacity (Koo et al., 2019). For example, bacteria such as *Bacteroides vulgatus*, which increased post-treatment, contributed to both reductions and increases in key pathways, reflecting the duality of their functional impacts on the microbiome. Conversely, the depletion of *Bifidobacterium bifidum*, critical for glycogen degradation, underscores the risks posed by the loss of beneficial strains. Moreover, our findings indicate that functional contributions stem not only from bacterial species with altered strains but also from those whose strains remained stable in abundance. For example, pathways such as thiamine biosynthesis and O-antigen building blocks biosynthesis were influenced by bacteria like *Bifidobacterium adolescentis*, *Faecalibacterium prausnitzii*, and *Roseburia faecis*, which remained relatively stable in their abundances post-antibiotic treatment. However, despite their stable population levels, changes in the surrounding microbial community likely altered their relative functional contributions. Such dynamics suggest that stable taxa can adapt their metabolic outputs in response to environmental changes, including those induced by antibiotic exposure.

We observed reduced viral diversity in donor samples following antibiotic exposure, further underscoring the disruption of the gut microbial ecosystem. Viruses, particularly bacteriophages, play an integral role in shaping bacterial fitness and maintaining gut homeostasis (Lam et al., 2022; Norman et al., 2015). Our findings demonstrated a decrease in alpha diversity of viruses in all donors post-antibiotic treatment, with a particularly pronounced reduction observed in Donor 2. Such reductions may reflect a diminished ability of the virome to regulate bacterial populations. Notably, the sustained rise in *crAssphage* abundance post-antibiotic treatment is of particular interest. *CrAssphage* is a dominant bacteriophage in the human gut microbiota, and studies have shown its association with stable and healthy microbiome in balanced proportions (Remesh and Viswanathan, 2024). However, in our study, its significant increase in Donor 1 and Donor 2 post-antibiotic treatment may also reflect potential disruption of microbial balance. *CrAssphage* relies on specific host bacteria for replication, and its sustained high levels, even 8 months or more after antibiotic exposure, indicate that recovery of the virome to a pre-treatment state may be incomplete. This persistence suggests a shift in host-bacteriophage dynamics, potentially driven by reduced bacterial diversity or altered ecological niches created by antibiotic disruption. Moreover, disproportionate *crAssphage* abundance has been linked to various diseases such as inflammatory bowel disease, obesity, metabolic syndrome, and colorectal cancer (Norman et al., 2015; Gogokhia et al., 2019; Cervantes-Echeverría et al., 2023). Viral components may contribute to FMT efficacy (Zuo et al., 2018; Fujimoto et al., 2021), but the clinical implications of virome/phageome changes in FMT donors remain unclear. It should be noted that the virome analysis in this study is limited to DNA. As a result, RNA viruses were not captured, which likely contributed to the low number of detected viral species. Future studies incorporating RNA sequencing methodologies, such as metatranscriptomics, would be necessary to provide a more comprehensive characterization of the gut virome.

One critical aspect of FMT safety is the transmission of antibiotic-resistant organisms (van Lingen, 2023; Food and Drug Administration, 2020; DeFilipp et al., 2019). These concerns underscore the broader challenge of antimicrobial resistance, where ARGs, capable of horizontal transfer, not only persist but actively reshape microbial ecosystems (Sabtu et al., 2015; Murray, 2022; Centers for Disease Control and Prevention, 2019). Our findings suggest that shifts in ARG diversity reflect the lasting impact of antibiotic exposure (Jernberg et al., 2007; Jakobsson et al., 2010; Xu et al., 2020), potentially altering the competitive dynamics of gut bacteria like *Bacteroides massiliensis* and *Bifidobacterium longum,* which were positively correlated with ARGs. While these correlations hint at possible ARG acquisition, they do not necessarily imply that these bacteria have become antibiotic-resistant. Fortunately, these changes did not involve the most hazardous ARGs, classified as “Rank I ARGs: current threats” (Zhang et al., 2021). Nevertheless, how these ARGs interacts with a dysbiotic microbial population remains incompletely understood.

Our study has several strengths. First, we performed longitudinal sampling of three long term stool donors with high efficacy in preventing rCDI. Second, we undertook deep shotgun metagenomics sequencing which enabled us to address many questions. Our study also has several limitations, including a small number of donors, different antibiotic regimens and durations for each donor, and varying durations of follow up. We did not capture success rates of recipients once these donors came out of quarantine in order to examine how these changes may impact efficacy. Although we profiled ARGs, we did not examine antibiotic resistant organisms in these stool donors.

In conclusion, while our sample size is limited, we found no increased risk of potential ARGs dissemination from donors to recipients after antibiotic exposure of donors, as assessed by the donor’s ARG profiles. The alterations in ARG profiles were minimal and do not raise major safety concerns for FMT. However, the impact of antibiotic exposure on donor microbiota was more pronounced than anticipated, with lasting and complex changes, particularly in key bacterial species and their functionality. These changes could potentially affect the efficacy of FMT, especially in indications beyond rCDI, and suggest that donor microbiota performance post-antibiotic treatment may be unpredictable. Therefore, donor suspension periods may need to be individualized, taking into account the spectrum of antibiotics, treatment duration, and other host-specific factors. Future studies should consider a broader range of antibiotics, varying treatment durations, and more frequent sampling, alongside additional host-specific factors like diet, lifestyle, and the use of additional medications like Proton Pump Inhibitors (PPIs) or probiotics. This comprehensive approach will help better understand the long-term effects of antibiotics on the gut microbiome and inform stool banks which is evidence-based.

## Data Availability

The metagenomic sequencing data from this study has been processed and deposited at NCBI, accessible under project ID PRJNA1202717, https://www.ncbi.nlm.nih.gov/bioproject/1202717. The custom scripts used for data analysis in this study are available in a GitHub repository, https://github.com/ramin-karimian/fmt_pj.

## Footnotes

^1^ https://huttenhower.sph.harvard.edu/kneaddata

^2^mpa_v30_CHOCOPhlAn_201901.

^3^ http://scikit-bio.org/
