# Supplementary material for: Prolonged effect of antibiotic therapy on the gut microbiota composition, functionality, and antibiotic resistance genes’ profiles in healthy stool donors
Source: Front Microbiol. 2025 May 9;16:1589704. doi: 10.3389/fmicb.2025.1589704 (PMC12098650; doi:10.3389/fmicb.2025.1589704)
Supplement: Supplementary file 1 [file Supplementary_file_1.zip › Supplementary Figures.PDF]

Supplementary Figures

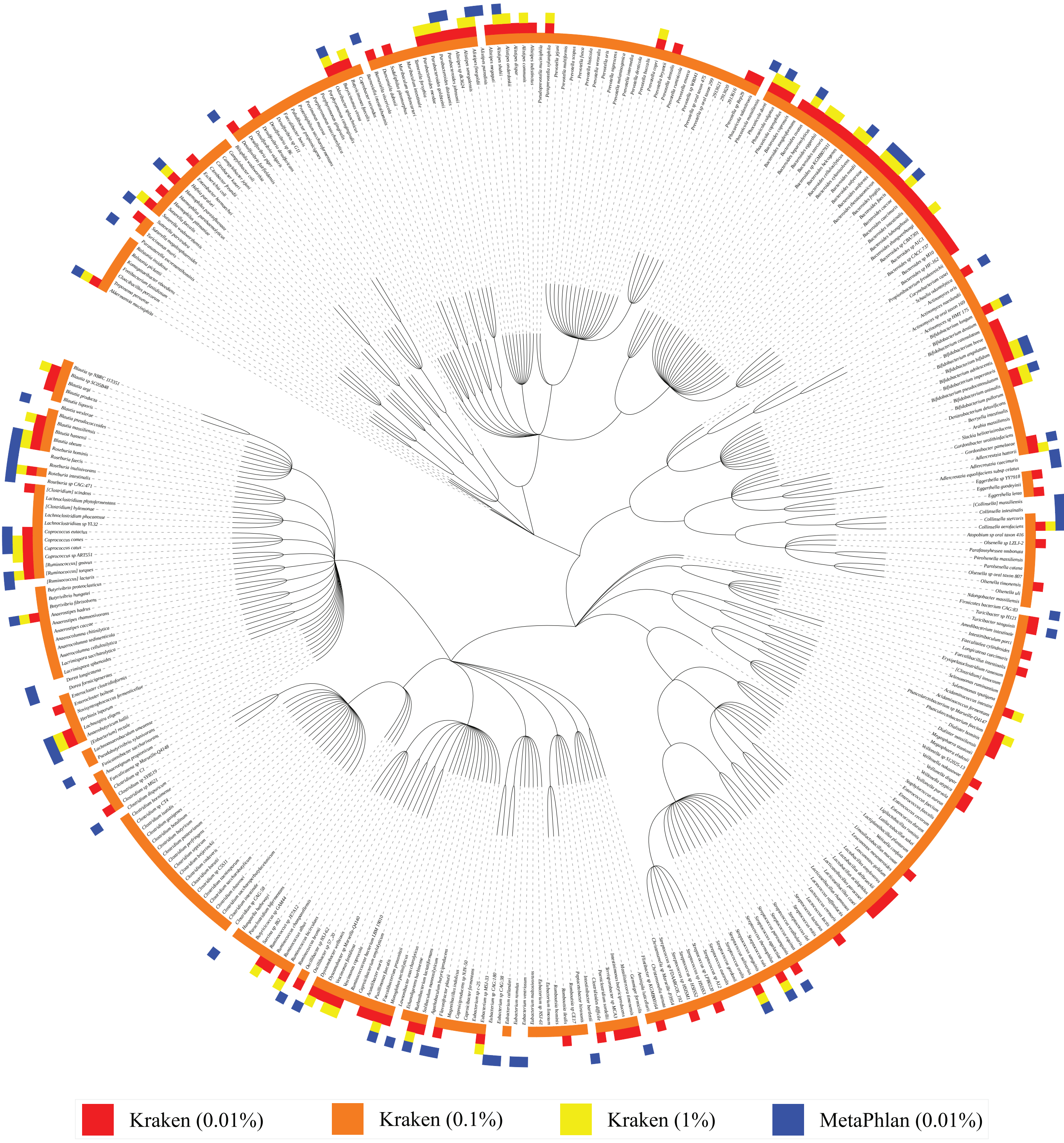

Kraken (0.01%)
Kraken (0.1%)
Kraken (1%)
MetaPhlan (0.01%)

Supplementary Figure S1. Phylogenetic tree for demonstration of the species coverage by Kraken (with 1%, 0.1%, and 0.01% filtering thresholds) and MetaPhlan3 (with 0.01% filtering threshold) pipelines.

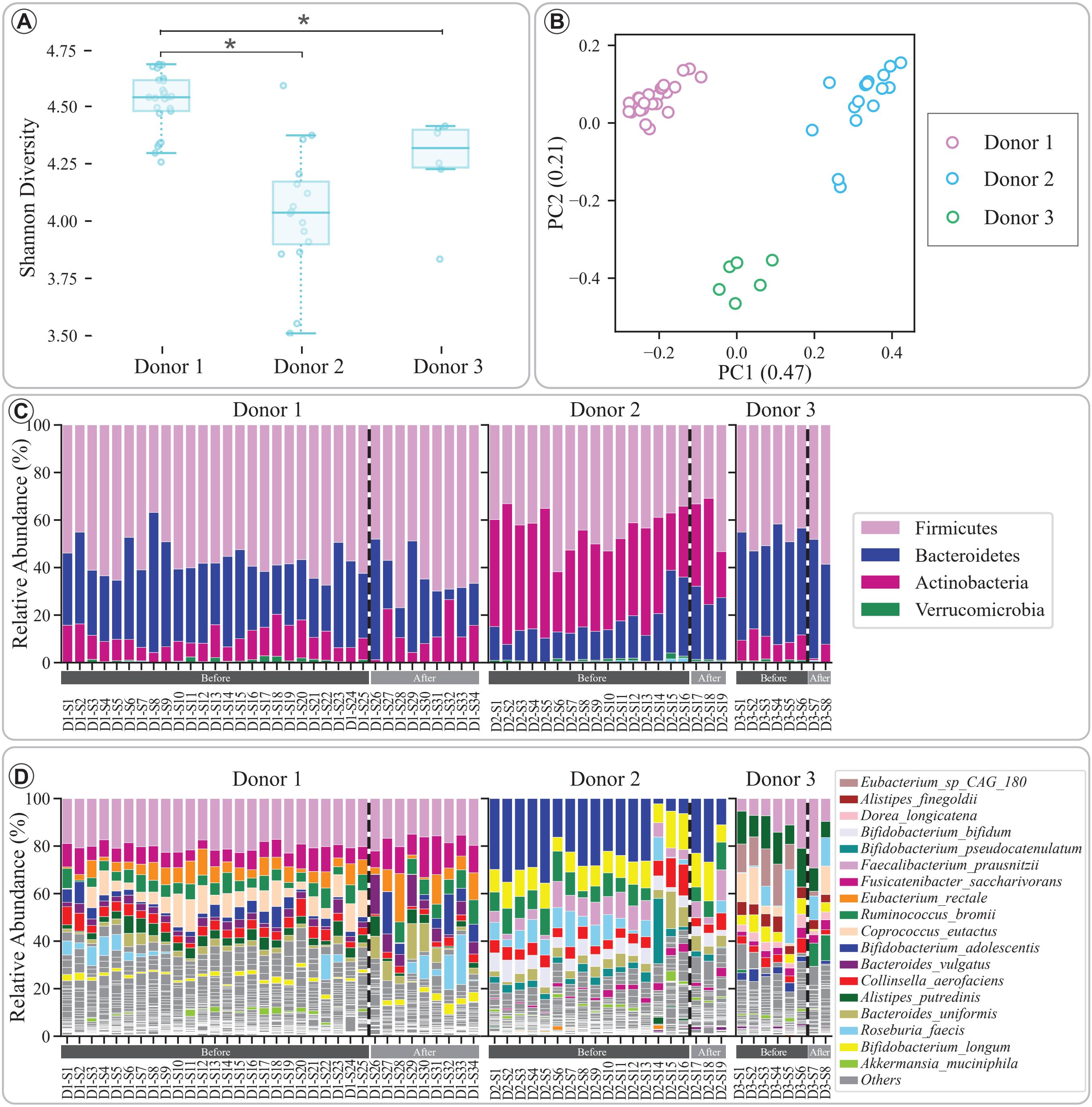
A
B
C
D
Shannon Diversity
Donor 1
Donor 2
Donor 3
PC1 (0.47)
PC2 (0.21)
Relative Abundance (%)
Before
After
Firmicutes
Bacteroidetes
Actinobacteria
Verrucomicrobia
Eubacterium_sp_CAG_180
Alistipes_finegoldii
Dorea_longicatena
Bifidobacterium_bifidum
Bifidobacterium_pseudocatenulatum
Faecalibacterium_prausnitzii
Fusicatenibacter_saccharivorans
Eubacterium_rectale
Ruminococcus_bromii
Coprococcus_eutactus
Bifidobacterium_adolescentis
Bacteroides_vulgatus
Collinsella_aerofaciens
Alistipes_putredinis
Bacteroides_uniformis
Roseburia_faecis
Bifidobacterium_longum
Akkermansia_muciniphila
Others

Supplementary Figure S2. Bacterial composition of samples from Donors 1, 2, and 3. (A) Comparison of the Shannon diversity index for samples from donors prior to antibiotic treatment (* indicates a significant change with a p-value < 0.05). (B) Evaluation of the distinct beta-diversity among the donors' samples taken before antibiotic treatment, visualized using PCoA plots based on the Bray-Curtis dissimilarity metric (Pairwise PERMANOVA test with FDR correction, adjusted p-value < 0.01). (C) Stacked bar plots depicting bacterial compositions at the phylum level. Before and after treatment samples are differentiated by a dashed line. The samples are presented in chronological order in this figure (D) Stacked bar plots illustrating bacterial compositions at the species level. Highly abundant bacterial species are colored differently, while others are shown in gray. Before and after treatment samples are separated by a dashed line. The samples are presented in chronological order in this figure.

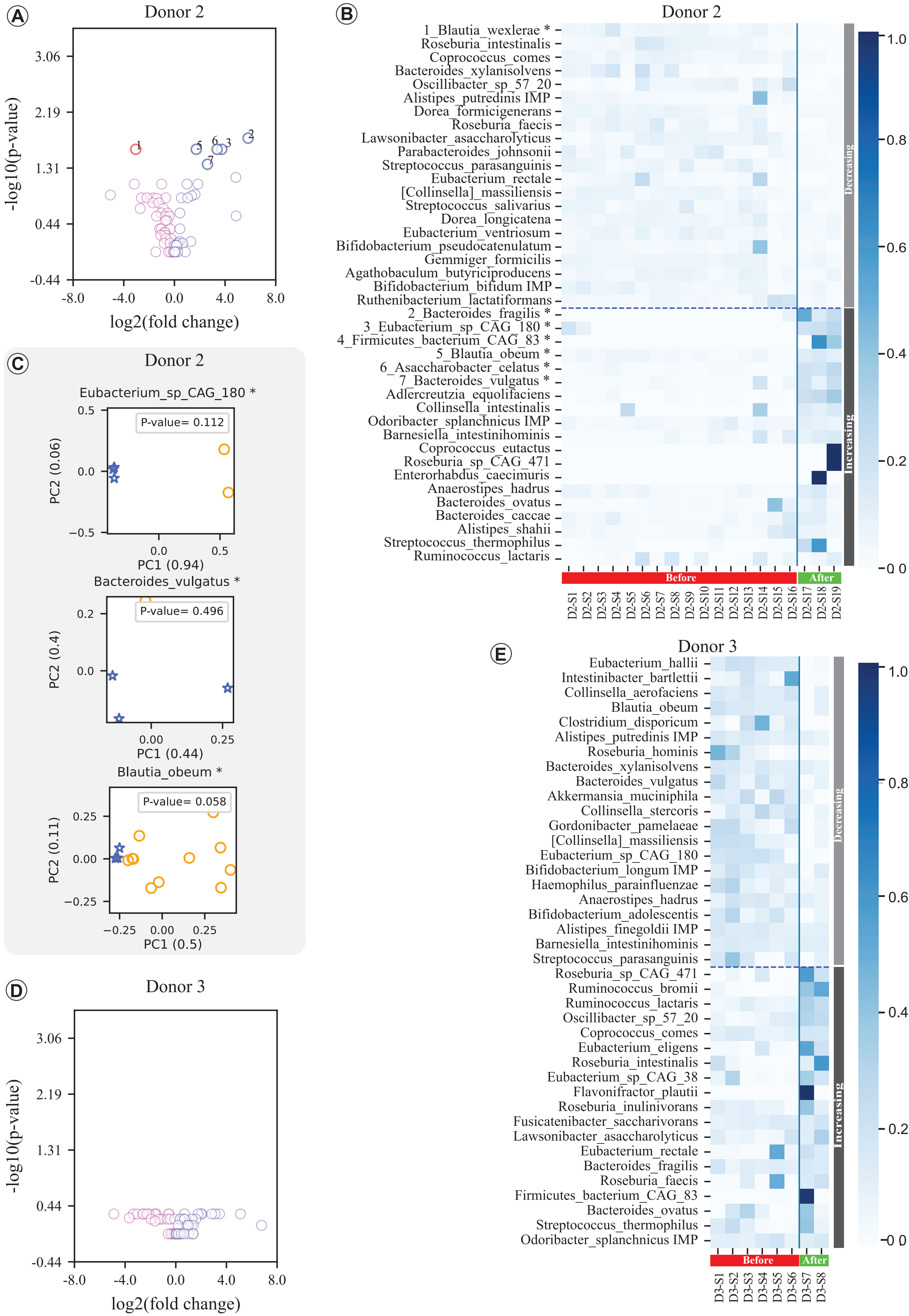

A
Donor 2
-log10(p-value)
log2(fold change)
B
Donor 2
1_Blautia_wexlerae *
Roseburia_intestinalis
Coprococcus_comes
Bacteroides_xylanisolvens
Oscillibacter_sp_57_20
Alistipes_putredinis IMP
Dorea_formicigenerans
Roseburia_faecis
Lawsonibacter_asaccharolyticus
Parabacteroides_johnsonii
Streptococcus_parasanguinis
Eubacterium_rectale
[Collinsella]_massiliensis
Streptococcus_salivarius
Dorea_longicatena
Eubacterium_ventriosum
Bifidobacterium_pseudocatenulatum
Gemmiger_formicilis
Agathobaculum_butyriciproducens
Bifidobacterium_bifidum IMP
Ruthenibacterium_lactatiformans
2_Bacteroides_fragilis *
3_Eubacterium_sp_CAG_180 *
4_Firmicutes_bacterium_CAG_83 *
5_Blautia_obeum *
6_Asaccharobacter_celatus *
7_Bacteroides_vulgatus *
Adlercreutzia_equolifaciens
Collinsella_intestinalis
Odoribacter_splanchnicus IMP
Barnesiella_intestinihominis
Coprococcus_eutactus
Roseburia_sp_CAG_471
Enterorhabdus_caecimuris
Anaerostipes_hadrus
Bacteroides_ovatus
Bacteroides_caccae
Alistipes_shahii
Streptococcus_thermophilus
Ruminococcus_lactaris
Decreasing
Increasing
Before
After
C
Donor 2
Eubacterium_sp_CAG_180 *
P-value= 0.112
PC2 (0.06)
PC1 (0.94)
Bacteroides_vulgatus *
P-value= 0.496
PC2 (0.4)
PC1 (0.44)
Blautia_obeum *
P-value= 0.058
PC2 (0.11)
PC1 (0.5)
D
Donor 3
-log10(p-value)
log2(fold change)
E
Donor 3
Eubacterium_hallii
Intestinibacter_bartlettii
Collinsella_aerofaciens
Blautia_obeum
Clostridium_disporicum
Alistipes_putredinis IMP
Roseburia_hominis
Bacteroides_xylanisolvens
Bacteroides_vulgatus
Akkermansia_muciniphila
Collinsella_stercoris
Gordonibacter_pamelaeae
[Collinsella]_massiliensis
Eubacterium_sp_CAG_180
Bifidobacterium_longum IMP
Haemophilus_parainfluenzae
Anaerostipes_hadrus
Bifidobacterium_adolescentis
Alistipes_finegoldii IMP
Barnesiella_intestinihominis
Streptococcus_parasanguinis
Roseburia_sp_CAG_471
Ruminococcus_bromii
Ruminococcus_lactaris
Oscillibacter_sp_57_20
Coprococcus_comes
Eubacterium_eligens
Roseburia_intestinalis
Eubacterium_sp_CAG_38
Flavonifractor_plautii
Roseburia_inulinivorans
Fusicatenibacter_saccharivorans
Lawsonibacter_asaccharolyticus
Eubacterium_rectale
Bacteroides_fragilis
Roseburia_faecis
Firmicutes_bacterium_CAG_83
Bacteroides_ovatus
Streptococcus_thermophilus
Odoribacter_splanchnicus IMP
Decreasing
Increasing
Before
After

Supplementary Figure S3. Effect of antibiotic treatment on bacteriome profile of Donors 2 and 3. (A) Volcano plot displaying bacterial species' response to treatment for donor 2. Magnitude of change is shown on the x-axis, and significance of change on the y-axis (Bacteria with p-values < 0.05, determined by Mann-Whitney test, are numbered in the figure). (B) Heatmap illustrating dynamics of the top 40 bacteria (sorted by raw p-values) in donor 2's samples, with horizontal dashed line separating decreasing and increasing bacteria, and solid vertical line distinguishing before and after treatment samples (* indicates significant change with FDR-adjusted p-value < 0.05, Mann-Whitney test). The samples are presented in chronological order in this figure. (C) PCA plots of strain communities with significant change post-treatment, using samples meeting the StrainPhlan pipeline's minimum thresholds (details in Methods). Circles and stars denote pre- and post-treatment samples, respectively. (D) Volcano plot displaying bacterial species' response to treatment for donor 3. The x-axis shows $\log_2$ change in feature abundances before vs. after treatment; the y-axis shows $-\log_{10}$ of the adjusted p-value. Significant changes (FDR-adjusted p-value < 0.05, Mann-Whitney) are indicated in the figure. (E) Heatmap illustrating dynamics of the top 40 bacteria (sorted by raw p-values) in donor 3's samples, with horizontal dashed line separating decreasing and increasing bacteria, and solid vertical line distinguishing before and after treatment samples. The samples are presented in chronological order in this figure.

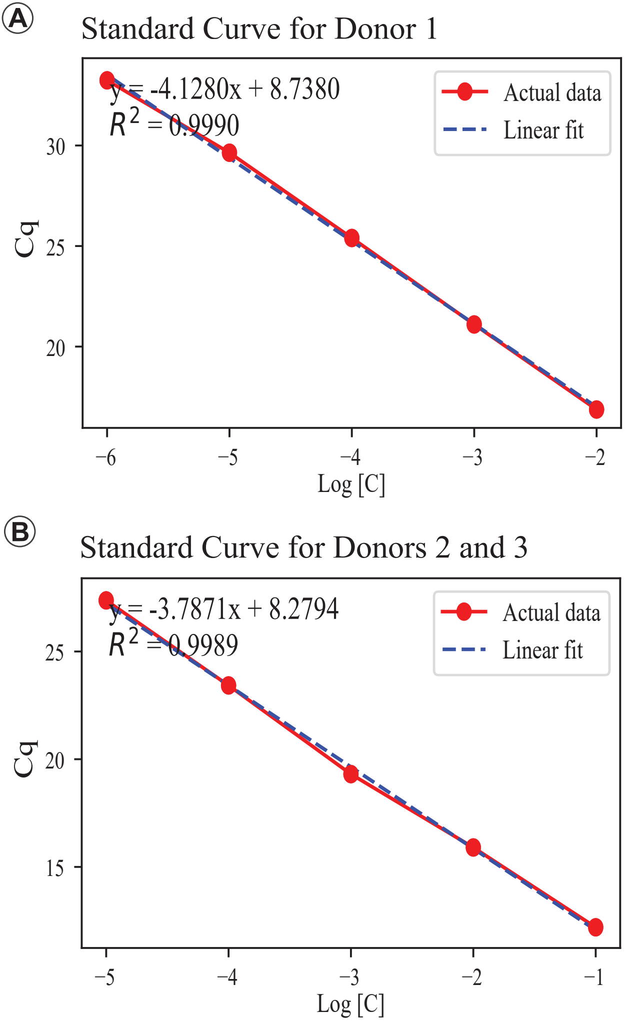
A
Standard Curve for Donor 1
y = -4.1280x + 8.7380
R^2 = 0.9990
Actual data
Linear fit
Cq
30
25
20
−6
−5
−4
−3
−2
Log [C]
B
Standard Curve for Donors 2 and 3
y = -3.7871x + 8.2794
R^2 = 0.9989
Actual data
Linear fit
Cq
25
20
15
−5
−4
−3
−2
−1
Log [C]

Supplementary Figure S4. Standard Curves for qPCR Assays of Donors 1, 2, and 3. (A) Donor 1: Slope of -3.7871 and R-squared value of 0.9989, indicating near-ideal amplification efficiency and robust assay performance. (B) Donors 2 and 3: Consistent slopes of -4.1273 and R-squared values of 0.9989, demonstrating reliable data fit and respectable amplification efficiency.

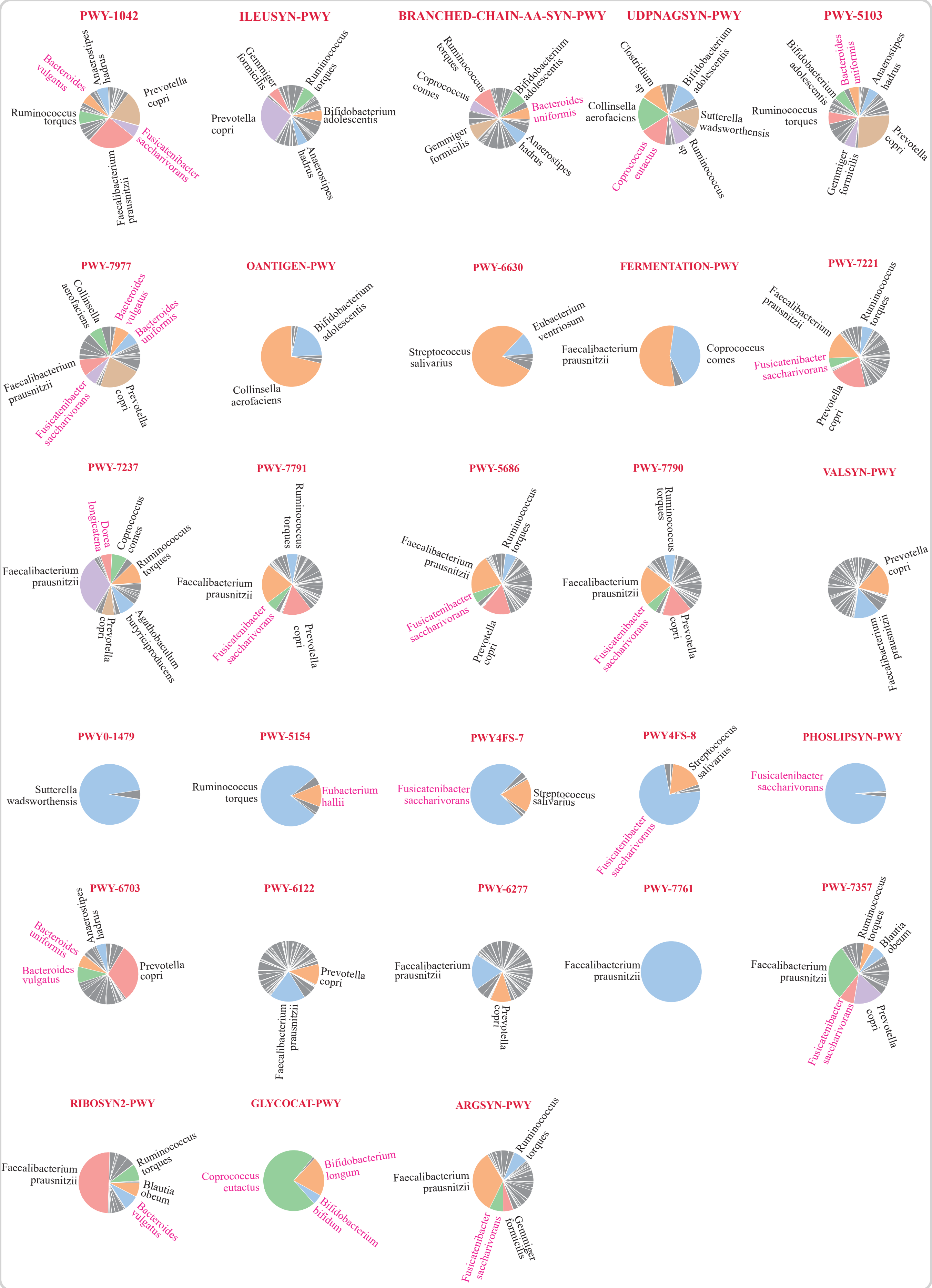

PWY-1042
Bacteroides vulgatus
Anaerostipes hadrus
Prevotella copri
Ruminococcus torques
Fusicatenibacter saccharivorans
Faecalibacterium prausnitzii
ILEUSYN-PWY
Gemmiger formicilis
Ruminococcus torques
Prevotella copri
Bifidobacterium adolescentis
Anaerostipes hadrus
BRANCHED-CHAIN-AA-SYN-PWY
Ruminococcus torques
Coprococcus comes
Bifidobacterium adolescentis
Bacteroides uniformis
Gemmiger formicilis
Anaerostipes hadrus
UDPNAGSYN-PWY
Clostridium sp
Bifidobacterium adolescentis
Collinsella aerofaciens
Sutterella wadsworthensis
Coprococcus eutactus
Ruminococcus sp
PWY-5103
Bifidobacterium adolescentis
Bacteroides uniformis
Anaerostipes hadrus
Ruminococcus torques
Prevotella copri
Gemmiger formicilis
PWY-7977
Collinsella aerofaciens
Bacteroides vulgatus
Bacteroides uniformis
Faecalibacterium prausnitzii
Fusicatenibacter saccharivorans
Prevotella copri
OANTIGEN-PWY
Bifidobacterium adolescentis
Collinsella aerofaciens
PWY-6630
Eubacterium ventriosum
Streptococcus salivarius
FERMENTATION-PWY
Faecalibacterium prausnitzii
Coprococcus comes
PWY-7221
Faecalibacterium prausnitzii
Ruminococcus torques
Fusicatenibacter saccharivorans
Prevotella copri
PWY-7237
Dorea longicatena
Coprococcus comes
Ruminococcus torques
Faecalibacterium prausnitzii
Prevotella copri
Agathobaculum butyriciproducens
PWY-7791
Ruminococcus torques
Faecalibacterium prausnitzii
Fusicatenibacter saccharivorans
Prevotella copri
PWY-5686
Faecalibacterium prausnitzii
Ruminococcus torques
Fusicatenibacter saccharivorans
Prevotella copri
PWY-7790
Ruminococcus torques
Faecalibacterium prausnitzii
Fusicatenibacter saccharivorans
Prevotella copri
VALSYN-PWY
Prevotella copri
Faecalibacterium prausnitzii
PWY0-1479
Sutterella wadsworthensis
PWY-5154
Ruminococcus torques
Eubacterium hallii
PWY4FS-7
Fusicatenibacter saccharivorans
Streptococcus salivarius
PWY4FS-8
Streptococcus salivarius
Fusicatenibacter saccharivorans
PHOSLIPSYN-PWY
Fusicatenibacter saccharivorans
PWY-6703
Bacteroides uniformis
Anaerostipes hadrus
Prevotella copri
Bacteroides vulgatus
PWY-6122
Prevotella copri
Faecalibacterium prausnitzii
PWY-6277
Faecalibacterium prausnitzii
Prevotella copri
PWY-7761
Faecalibacterium prausnitzii
PWY-7357
Ruminococcus torques
Blautia obeum
Faecalibacterium prausnitzii
Fusicatenibacter saccharivorans
Prevotella copri
RIBOSYN2-PWY
Faecalibacterium prausnitzii
Ruminococcus torques
Blautia obeum
Bacteroides vulgatus
GLYCOCAT-PWY
Coprococcus eutactus
Bifidobacterium longum
Bifidobacterium bifidum
ARGSYN-PWY
Ruminococcus torques
Faecalibacterium prausnitzii
Fusicatenibacter saccharivorans
Gemmiger formicilis

Supplementary Figure S5. Pie charts depicting the significantly decreased pathways in donor 1 are presented, showcasing the respective bacterial contributions. Bacteria contributing less than 5% are depicted in gray and remain unlabeled within the figure. In contrast, bacteria of significance in the context of FMT engraftment or those that have undergone significant changes are labeled in pink. It should be noted that pie charts are shown only for pathways with at least one contributing bacterium reported by the HUMAnN3 pipeline (i.e., excluding those with only 'unclassified' contributors).

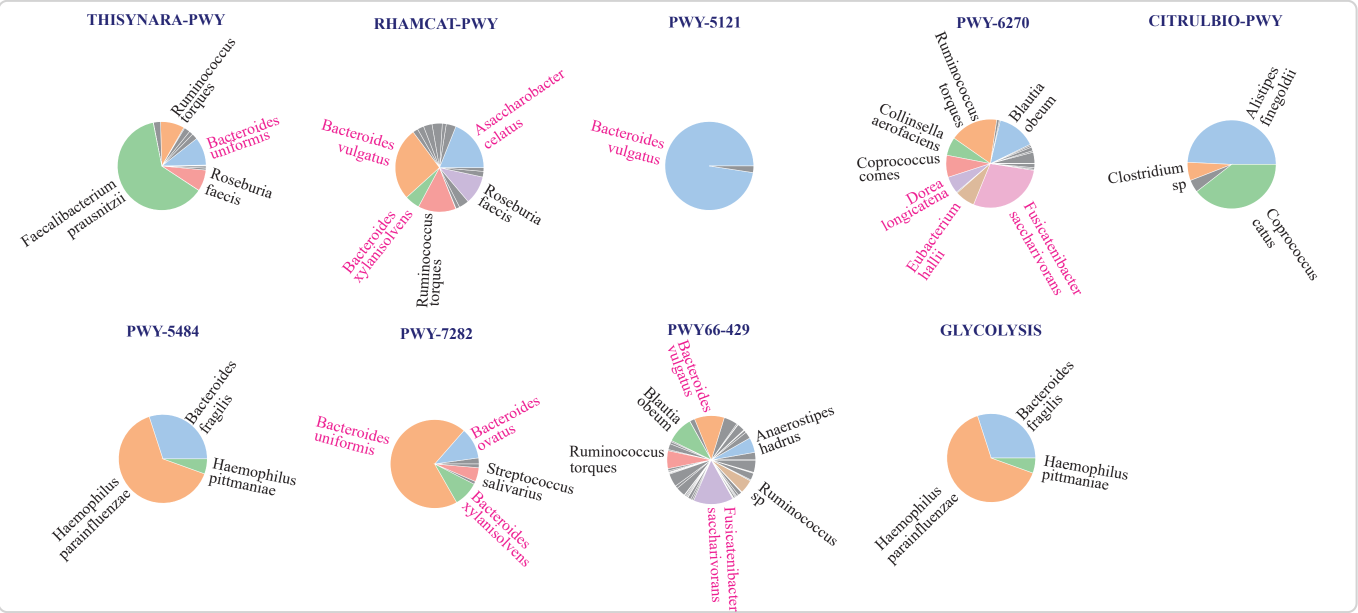

THISYNARA-PWY
Ruminococcus torques
Bacteroides uniformis
Roseburia faecis
Faecalibacterium prausnitzii
RHAMCAT-PWY
Asaccharobacter celatus
Bacteroides vulgatus
Roseburia faecis
Bacteroides xylanisolvens
Ruminococcus torques
PWY-5121
Bacteroides vulgatus
PWY-6270
Ruminococcus torques
Blautia obeum
Collinsella aerofaciens
Coprococcus comes
Dorea longicatena
Eubacterium hallii
Fusicatenibacter saccharivorans
CITRULBIO-PWY
Alistipes finegoldii
Clostridium sp
Coprococcus catus
PWY-5484
Bacteroides fragilis
Haemophilus pittmaniae
Haemophilus parainfluenzae
PWY-7282
Bacteroides uniformis
Bacteroides ovatus
Streptococcus salivarius
Bacteroides xylanisolvens
PWY66-429
Bacteroides vulgatus
Blautia obeum
Anaerostipes hadrus
Ruminococcus torques
Ruminococcus sp
Fusicatenibacter saccharivorans
GLYCOLYSIS
Bacteroides fragilis
Haemophilus pittmaniae
Haemophilus parainfluenzae

Supplementary Figure S6. Pie charts depicting the significantly increased pathways in donor 1 are presented, showcasing the respective bacterial contributions. Bacteria contributing less than 5% are depicted in gray and remain unlabeled within the figure. In contrast, bacteria of significance in the context of FMT engraftment or those that have undergone significant changes are labeled in pink. It should be noted that pie charts are shown only for pathways with at least one contributing bacterium reported by the HUMAnN3 pipeline (i.e., excluding those with only 'unclassified' contributors).
