# Supplementary material for: Prolonged effect of antibiotic therapy on the gut microbiota composition, functionality, and antibiotic resistance genes’ profiles in healthy stool donors
Source: Front Microbiol. 2025 May 9;16:1589704. doi: 10.3389/fmicb.2025.1589704 (PMC12098650; doi:10.3389/fmicb.2025.1589704)
Supplement: Supplementary file 1 [file Supplementary_file_1.zip › Supplementary Tables.PDF]

Supplementary Tables

Supplementary Table S1. Primers, probes, and cycling conditions of qPCR assays [1].

Marker gene	Primers and probes	Primer and probe concentration	Cycling parameters
**CPQ_056**	Forward:<br>CAGAAGTACAAACTCCTAAAAAACGTAGAG<br>Reverse:<br>GATGACCAATAAACAAGCCATTAGC<br>Probe:<br>[FAM] AATAACGATTTACGTGATGTAAC [MGB]	1uM<br>1uM<br>80nM	5 min at 95 °C, followed by 35 cycles of 5 s at 95 °C, and 60 s at 60 °C

Supplementary Table S2. The list of top20 highly engrafted bacteria in FMT [2-5].

TAXONOMY ID	BACTERIA
165179	*Bifidobacterium bifidum*
338188	*Alistipes putredinis*
28117	*Odoribacter splanchnicus*
214856	*Phascolarctobacterium faecium*
204516	*Sutterella wadsworthensis*
84112	*Bacteroides stercoris*
1681	*Eggerthella lenta*
216816	*Prevotella copri*
487173	*Phocaeicola massiliensis*
216572	*Alistipes finegoldii*
2292349	*Oscillospiraceae*
572010	*Bifidobacterium longum*
2292991	*Bacteroides finegoldii*
46506	*Paraprevotella clara*
28118	*Dialister succinatiphilus*
454154	*Parasutterella excrementihominis*
40545	*Slackia isoflavoniconvertens*
33025	*Clostridia bacterium*
487175	*Eubacterium sp. AM28-29*
2044939	*Catenibacterium sp. AM22-15*

2

Supplementary Table S3. Statistics on the number of unique species identified by each pipeline and at each filtering threshold, applied to remove the low abundance taxa.

Pipeline	Kraken	Kraken	Kraken	MetaPhlan
Filtering Threshold	1%	0.1%	0.01%	0.01%
Number of Species	60	142	328	72

Supplementary Table S4. Detailed information regarding the significantly altered pathways in Donor 1.

IN FIGURE #	REF ID	ACTUAL NAME	SUPERCLASS
**1**	PWY-1042	glycolysis IV	Generation of Precursor Metabolites and Energy
**2**	ILEUSYN-PWY	L-isoleucine biosynthesis I (from threonine)	Amino Acid Biosynthesis
**3**	BRANCHED-CHAIN-AA-SYN-PWY	superpathway of branched chain amino acid biosynthesis	Amino Acid Biosynthesis
**4**	UDPNAGSYN-PWY	UDP-N-acetyl-D-glucosamine biosynthesis I	Carbohydrate Biosynthesis
**5**	PWY-5103	L-isoleucine biosynthesis III	Amino Acid Biosynthesis
**6**	PWY-7977	L-methionine biosynthesis IV	Amino Acid Biosynthesis
**7**	OANTIGEN-PWY	O-antigen building blocks biosynthesis (E. coli)	Carbohydrate Biosynthesis
**8**	PWY-6630	superpathway of L-tyrosine biosynthesis	Amino Acid Biosynthesis
**9**	FERMENTATION-PWY	mixed acid fermentation	Fermentation
**10**	PWY-7221	guanosine ribonucleotides de novo biosynthesis	Nucleoside and Nucleotide Biosynthesis
**11**	PWY-7237	myo-, chiro- and scyllo-inositol degradation	Degradation/Utilization/Assimilation
**12**	PWY-7791	UMP biosynthesis III	Nucleoside and Nucleotide Biosynthesis
**13**	PWY-5686	UMP biosynthesis I	Nucleoside and Nucleotide Biosynthesis
**14**	PWY-7790	UMP biosynthesis II	Nucleoside and Nucleotide Biosynthesis
**15**	VALSYN-PWY	L-valine biosynthesis	Amino Acid Biosynthesis
**16**	PWY0-1479	tRNA processing	Nucleic Acid Processing
**17**	PWY-5154	L-arginine biosynthesis III (via N-acetyl-L-citrulline)	Amino Acid Biosynthesis
**18**	PWY4FS-7	phosphatidylglycerol biosynthesis I (plastidic)	Fatty Acid and Lipid Biosynthesis

**19**	PWY4FS-8	phosphatidylglycerol biosynthesis II (non-plastidic)	Fatty Acid and Lipid Biosynthesis
**20**	PHOSLIPSYN-PWY	superpathway of phospholipid biosynthesis I (bacteria)	Fatty Acid and Lipid Biosynthesis
**21**	PWY-6703	preQ0 biosynthesis	Secondary Metabolite Biosynthesis
**22**	PWY-6122	5-aminoimidazole ribonucleotide biosynthesis II	Nucleoside and Nucleotide Biosynthesis
**23**	PWY-6277	superpathway of 5-aminoimidazole ribonucleotide biosynthesis	Nucleoside and Nucleotide Biosynthesis
**24**	PWY-7761	NAD salvage pathway II (PNC IV cycle)	Cofactor, Carrier, and Vitamin Biosynthesis
**25**	PWY-7357	thiamine diphosphate formation from pyrithiamine and oxythiamine (yeast)	Cofactor, Carrier, and Vitamin Biosynthesis
**26**	RIBOSYN2-PWY	flavin biosynthesis I (bacteria and plants)	Cofactor, Carrier, and Vitamin Biosynthesis
**27**	GLYCOCAT-PWY	glycogen degradation I	Carbohydrate Degradation
**28**	FOLSYN-PWY	superpathway of tetrahydrofolate biosynthesis and salvage	Cofactor, Carrier, and Vitamin Biosynthesis
**29**	ARGSYN-PWY	L-arginine biosynthesis I (via L-ornithine)	Amino Acid Biosynthesis
**30**	THISYNARA-PWY	isoprene biosynthesis I	Secondary Metabolite Biosynthesis
**31**	RHAMCAT-PWY	glycolysis II (from fructose 6-phosphate)	Generation of Precursor Metabolites and Energy
**32**	PWY-5121	peptidoglycan maturation (meso-diaminopimelate containing)	Cell Structure Biosynthesis
**33**	PWY-6270	homolactic fermentation	Fermentation
**34**	CITRULBIO-PWY	L-citrulline biosynthesis	Amino Acid Biosynthesis
**35**	PWY-7392	taxadiene biosynthesis (engineered)	Secondary Metabolite Biosynthesis
**36**	PWY-5484	pyrimidine deoxyribonucleosides salvage	Nucleoside and Nucleotide Biosynthesis

**37**	PWY-7282	superpathway of pyrimidine deoxyribonucleotides de novo biosynthesis	Nucleoside and Nucleotide Biosynthesis
**38**	ANAEROFRUCAT-PWY	4-amino-2-methyl-5-diphosphomethylpyrimidine biosynthesis II	Cofactor, Carrier, and Vitamin Biosynthesis
**39**	PWY66-429	superpathway of geranylgeranyl diphosphate biosynthesis II (via MEP)	Polyprenyl OR Secondary Metabolite Biosynthesis
**40**	PWY-6895	glycogen degradation I	Carbohydrate Degradation
**41**	GLYCOLYSIS	chitin derivatives degradation	Carbohydrate Degradation
**42**	PWY-7211	L-valine biosynthesis	Amino Acid Biosynthesis
**43**	PWY-6906	chitin derivatives degradation	Carbohydrate Degradation

Supplementary Table S5. The full names of significantly changed ARGs in Donor 1 samples (the IDs match the numbers in Figure 4C).

ID	Short Name	Full Name
**1**	CfxA6	CfxA6
**2**	Hpyl_23S_CLR	Helicobacter pylori 23S rRNA with mutation conferring resistance to clarithromycin
**3**	Cpsi_16S_SPT	Chlamydophila psittaci 16S rRNA mutation conferring resistance to spectinomycin
**4**	Mgal_23S_PLM	Mycoplasma gallisepticum 23S rRNA mutation conferring resistance to pleuromutilin antibiotics
**5**	Mfer_23S_MAC	Mycoplasma fermentans 23S rRNA with mutation conferring resistance to macrolide antibiotics
**6**	Crei_16rrnS_STR	Chlamydomonas reinhardtii 16S rRNA mutation in the rrnS gene conferring resistance to streptomycin
**7**	Tthe_23S_PLM	Thermus thermophilus 23s rRNA conferring resistance to pleuromutilin antibiotics
**8**	Ctra_23S_MAC	Chlamydia trachomatis 23S rRNA with mutation conferring resistance to macrolide antibiotics
**9**	Mef(En2)	Mef(En2)
**10**	Mtub_rpoB_RIF	Mycobacterium tuberculosis rpoB mutants conferring resistance to rifampicin
**11**	Mhom_23S_MAC	Mycoplasma hominis 23S rRNA with mutation conferring resistance to macrolide antibiotic
**12**	Mche_16S_AMK	Mycobacteroides chelonae 16S rRNA mutation conferring resistance to amikacin
**13**	Mabs_16S_NEO	Mycobacteroides abscessus 16S rRNA mutation conferring resistance to neomycin
**14**	Bhyo_23S_TYL	Brachyspira hyodysenteriae 23S rRNA with mutation conferring resistance to tylosin
**15**	Msme_16rrsB_VIO	Mycolicibacterium smegmatis 16S rRNA mutation in the rrsB gene conferring resistance to viomycin
**16**	tet(W)	tetW
**17**	Mgen_23S_MULT	Mycoplasma genitalium 23S rRNA mutations confers resistance to fluoroquinolone and macrolide antibiotics
**18**	Mche_23S_CLR	Mycobacteroides chelonae 23S rRNA with mutation conferring resistance to clarithromycin
**19**	Mpne_23S_ERY	Mycoplasma pneumoniae 23S rRNA mutation conferring resistance to erythromycin
**20**	Ecol_rpoB_RIF	Escherichia coli rpoB mutants conferring resistance to rifampicin
**21**	Saur_fusA_FA	Staphylococcus mutant fusA gene conferring resistance to fusidic acid
**22**	Pmul_16S_SPT	Pasteurella multocida 16S rRNA mutation conferring resistance to spectinomycin

**23**	Mkan_23S_CLR	Mycobacterium kansasii 23S rRNA with mutation conferring resistance to clarithromycin
**24**	CblA-1	CblA-1

References

1. Stachler, E., et al., *Quantitative CrAssphage PCR assays for human fecal pollution measurement.* Environmental science & technology, 2017. **51**(16): p. 9146-9154.

2. Smith, B.J., et al., *Strain-resolved analysis in a randomized trial of antibiotic pretreatment and maintenance dose delivery mode with fecal microbiota transplant for ulcerative colitis.* Scientific reports, 2022. **12**(1): p. 5517.

3. Smillie, C.S., et al., *Strain tracking reveals the determinants of bacterial engraftment in the human gut following fecal microbiota transplantation.* Cell host & microbe, 2018. **23**(2): p. 229-240. e5.

4. Kootte, R.S., et al., *Improvement of insulin sensitivity after lean donor feces in metabolic syndrome is driven by baseline intestinal microbiota composition.* Cell metabolism, 2017. **26**(4): p. 611-619. e6.

5. Ianiro, G., et al., *Variability of strain engraftment and predictability of microbiome composition after fecal microbiota transplantation across different diseases.* Nature Medicine, 2022. **28**(9): p. 1913-1923.
